# Body image in children and adolescents diagnosed with the human immunodeficiency virus: a systematic review

**DOI:** 10.1590/1516-3180.2022.0154.R2.19082022

**Published:** 2022-11-21

**Authors:** Suellem Zanlorenci, Andressa Ferreira da Silva, Diego Augusto Santos Silva

**Affiliations:** I BSc. Master Student, Department of Physical Education, Universidade Federal de Santa Catarina (UFSC), Florianópolis (SC), Brazil.; II MSc. Doctoral Student, Department of Physical Education, Universidade Federal de Santa Catarina (UFSC), Florianópolis (SC), Brazil.; III MSc, PhD. Associate Professor, Department of Physical Education, Universidade Federal de Santa Catarina (UFSC), Florianópolis (SC), Brazil; and Associate Researcher, Faculty of Health Sciences, Universidad Autónoma de Chile, Providencia, Chile.; Universidad Autónoma de Chile, Faculty of Health Sciences, Providencia, Chile

**Keywords:** Body dissatisfaction, Body composition, Body image, Child health, Adolescent health, HIV, Body satisfaction, Children, Teenager

## Abstract

**CONTEXT:**

The relationship with body image, which is the way the body presents itself to each subject, can be aggravated in children and adolescents diagnosed with an human immunodeficiency virus (HIV) infection, since these patients use antiretroviral therapy and may suffer from the adverse effects of the treatment due to continuous use of medication.

**OBJECTIVE:**

To estimate the prevalence of body image dissatisfaction, to describe the assessment methods, and to identify associated factors in children and adolescents diagnosed with HIV.

**DESIGN AND SETTING:**

This is a systematic review. Department of Physical Education, Florianópolis - Brazil

**METHODS:**

We followed the procedures of the Preferred Reporting Items for Systematic Reviews (PRISMA) and the Cochrane recommendations in the selection of articles through a search performed in eight databases.

**RESULTS:**

Prevalence of body image dissatisfaction due to thinness was between 36.7–52.0% in males and 28.1–36.4% in females, and body image dissatisfaction due to overweight was between 8.0–31.2% in males and 21.9–50.0% in females. Factors associated with body image dissatisfaction were as follows: female sex, older age, low levels of physical activity, low self-esteem, higher body fat, higher body weight, greater arm muscle area, triceps skinfold thickness, and higher body mass index.

**CONCLUSION:**

Children and adolescents of both sexes diagnosed with HIV infection are dissatisfied by thinness and overweight of their body image.

**REGISTRATION:**

https://www.crd.york.ac.uk/prospero/ (CRD42021257676).

## INTRODUCTION

The bodily manifestations related to the human immunodeficiency virus (HIV) and its treatment with antiretroviral therapy (ART) negatively affect not only the physical but also the psychological health of children and adolescents diagnosed with HIV infection; including body image issues.^1–5^ Morphological changes related to lipodystrophy include loss of fat normally located in the face, buttocks, and extremities (lipoatrophy), contributing to perceived thinness, or gains of fat in the breasts, abdomen, and neck (lipohypertrophy), contributing to perceived overweight.^1,2,5,6^ Children and adolescents with HIV infection are at risk of dissatisfaction with their body image (i.e., the way the body is presented to each child).^3–5^ Furthermore, in children and adolescents without an HIV diagnosis, body image can be influenced by numerous physical, psychological, environmental, and cultural factors as determined subjectively by each child, and these may include the child's sex, age, media, beliefs, race, and general values, all of which also apply to children and adolescents diagnosed with HIV infection.^3–5^

Body image is a unique, dynamic, and multifaceted construction. Self-report body image assessment tools can take many forms, including questionnaires and scales with silhouettes, photos, or videos that represent stimuli with which respondents can compare and evaluate themselves.^7^ The choice of an assessment instrument by an investigator should take into account the age group that will be assessed, the nature of the assessment method, and the psychometric properties of the instrument (eg, reliability and validity for the population and uses for the investigator).^9^

As noted above, assessing the body image of children and adolescents diagnosed with HIV infection is made important by the types of symptoms and medication (i.e., continuous ART) side effects that may redefine body contours and self-perceptions of these patients.^3–5^ Conducting a systematic review can capture, recognize, and synthesize scientific evidence to support proposals for qualified health practices and implement evidence-based practice.^8^ In addition, the systematic review has a rigorous methodology proposed to identify studies on a topic in question, applying explicit and systematized search methods that assess the quality and validity of these studies.^8^ In this sense, with the absence of a cure for chronic diseases such as HIV infection, the study of body image in children and adolescents diagnosed with it can help to understand the subgroups most likely to be dissatisfied with their body image.^10^ Body image assessment tools can be critically important for directing the distribution of resources and implementing a variety of health programs to address physical, psychological, and social aspects of care for these patients.^11^

## OBJECTIVE

Our aims were to systematically review the existing scientific literature in this area to estimate the prevalence of dissatisfaction with body image, to describe the assessment methods, and to identify associated factors in children and adolescents diagnosed with HIV infection.

## METHODS

The report of this review is in accordance with the Preferred Reporting Items for Systematic Reviews (PRISMA)^12^ and follows the recommendations of the Cochrane Collaboration Handbook^13^ to answer the following question: what does the literature include about the prevalence, associated factors, and methods for assessing body image in children and adolescents diagnosed with HIV infection? The protocol for this study was registered in the PROSPERO database (registration number: CRD42021257676).

### Search strategy, descriptors and keywords

The search was performed in the following databases: 1) PubMed via National Library of Medicine (MEDLINE); 2) Web of Science; 3) Scopus; 4) SPORTDiscus via EBSCOhost; 5) LILACS via Virtual Health Library; 6) Scientific Electronic Library Online (SciELO); 7) PsycINFO via the American Psychological Association (APA); and 8) Cumulative Index to Nursing and Allied Health Literature (CINAHL), via EBSCOhost.

The search for articles in databases was performed using the advanced search tool, based on the construction of blocks of descriptors and keywords related to the theme. The selection of descriptors was performed by consulting the Medical Subject Headings (MeSH) and Descriptors in Health Sciences (DeCS)^14^ platforms related to the PECO acromion (patient/population, exposure, comparison, and outcome). Keywords were also selected through consensus in published sources (original articles). Depending on the database, keywords and descriptors were entered in Portuguese, English, and/or Spanish.

The first block (outcome) was composed of terms referring to body image, the second block was composed of the population of interest (children and adolescents), and the third block was composed of the term related to HIV (Appendix 1).

The “OR” Boolean operator was used to add at least one keyword or descriptor of each block in the advanced search and the “AND” operator to relate the blocks of keywords/descriptors to each other. In addition, quotation marks (“”) were used in compound words and to search for exact terms or expressions. Parentheses were used to combine search terms by outcome, exposure, and population categories. Asterisk (*) was used to search for all words derived from the same prefix.

The search was carried out in June 2021, considering all articles published up to this date. Additionally, the reference lists of eligible studies and those related to the topic of this review were manually searched to find possible relevant studies.

### Eligibility criteria

Inclusion criteria were as follows: (a) population composed of children and/or adolescents (aged 0–19 years or with average age of up to 19 years) with diagnosis of HIV infection; (b) cross-sectional, longitudinal case-control studies, cohort studies, interventions, or randomized clinical trials that allowed extracting information about the body image of children and adolescents diagnosed with HIV infection. The study had the following exclusion criteria: theses, dissertations, monographs, abstracts, book chapters, point of view and review articles, validation and/or reproducibility articles, articles to determine cutoff points, and articles that did not present data classifying individuals according to body image. However, these publications were screened (available text and references) to find complete articles of interest to this review.

### Selection of studies

Two independent reviewers (SZ and AFS) examined each database to obtain potential articles; duplicate articles were excluded and then other articles were excluded after the reading of titles and abstracts. Subsequently, the texts of selected articles were read in full for the selection of studies. A literature search was carried out in the references of the selected studies to select possible articles eligible for this review, not identified in the systematic search in databases. Disagreements between the two reviewers were resolved by a consensus meeting. A third reviewer (DASS) was consulted for unresolved disagreements.

The Zotero bibliographic manager version 5.0 (Roy Rosenzweig Center for History and New Media, Fairfax, Virginia, United States) was used to create specific libraries, which enabled the identification and exclusion of duplicate articles, and division and organization of the results of each database.

### Data extraction

Data were extracted by two independent reviewers (SZ and AFS) and consistency between them was checked by a third reviewer (DASS). The following information was extracted: names of authors, year of publication, methodological quality score, study site, age group investigated, population and sample, study design, stratification, test used to assess body image (example: scale of silhouettes, questionnaires, weight perception, etc.), prevalence, and associated factors.

### Risk of Bias

The risk of bias/methodological quality of selected articles was assessed by two independent researchers (SZ and AFS). For cases of disagreement between the two, the third researcher (DASS) with experience in systematic review was consulted through a consensus meeting. To assess the risk of bias, a tool proposed by the National Heart, Lung and Blood Institute (NIH)^15^ was used, according to the type of study. For cross-sectional and longitudinal studies, the Quality Assessment Tool for Observational Cohort and Cross-Sectional Studies was used. The Quality Assessment Tool for Observational Cohort and Cross-Sectional Studies (https://www.nhlbi.nih.gov/health-topics/study-quality-assessment-tools)^15^ is the recommended tool to assist in the assessment of internal validity (potential selection risk, information, measurement, or confounding factors) of cross-sectional and cohort studies.

Each question was scored with “0” or “1”, “0” being applied to questions answered with “no” and “1” for those answered with “yes” or “not applicable”. The “not applicable” option was used when it was not possible to evaluate one of the instrument's criteria due to the type of study (such as those with a cross-sectional design). The total score was obtained by summing the score of each question (https://www.nhlbi.nih.gov/health-topics/study-quality-assessment-tools).^15^

## RESULTS

**Figure 1:**
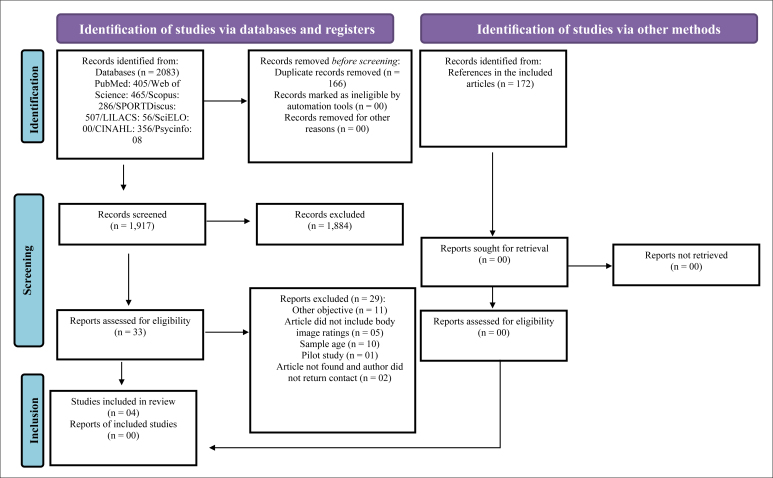
Flowchart of search, selection, and exclusion of articles.

In total, 2,083 articles were found; however, 166 were duplicates, resulting in 1,917 articles. After reading titles and abstracts, 1,884 studies were excluded because they did not meet the eligibility criteria, then 33 articles were read in full. Of these, four were included because they met the eligibility criteria.^3–5,16^ Subsequently, the references of included articles were read, but no new articles were included in this review (Figure 1).

Of the four included studies, three were carried out in Brazil^3–5^ and one in the United States of America.^16^ The population evaluated comprised a total of 455 individuals of both sexes. Of studies that used samples stratified by sex, there were a total of 89 females and 71 males,^3–5^ one study did not present stratification by sex.^16^ All studies had cross-sectional design^3–5,16^ (Table 1).

**Table 1:** Description of studies on body image in children and adolescents diagnosed with human immunodeficiency virus (HIV) infection

Author(s), place and year	Outline	Population/Sample	Age group	Viral Load/ART/Stage of the disease
“AIDS among children--United States, 1996.^16^ Division of HIV/AIDS Prevention, CDC”, New York, Florida, New Jersey, California, Puerto Rico and Texas, United States (1997)	Cross-sectional study*	295 adolescents	13 to 15 years	Viral Load: NR ART: NR Stage of the disease: NR
Alves Junior et al., ^3^ Florianópolis (SC), Brasil (2021)	Cross-sectional study	65 chidren and adolescents ♂: 30 ♀;: 35	8 to 15 years	Viral Load: Average ♂;: 2.2(1.0) Average ♀;:2.1 (0.9) ART: With Protease Inhibitor: ♂;: n = 19 (48.7%); ♀;: n = 20 (51.3%) Without Protease Inhibitor: ♂;: n = 06 (40.0%); ♀;: n = 09 (60.0%) Do not use: ♂;: n = 05 (45.5%); ♀;: n = 06 (54.5%) Disease stage: NR
Augustemak de Lima et al.,^4^ Florianópolis (SC), Brasil (2018)	Cross-sectional study	111 adolescents 57 living with HIV ♂;: 25 ♀;: 32 54 comparisons ♂;: 26 ♀;: 28	10 to 15 years	Viral charge: The absolute and relative count of CD4 + T lymphocytes was 791.3 cells.mm −3 e 30.4%, respectively (SD = 280.7 cells.mm −3 and 7.5%, respectively). An adolescent living with HIV had an undetectable viral load (< 40 copies.mL −1) ART: Inhibitor nucleoside analogue reverse transcriptase (NRTI): n = 49 (86.0%) Non-nucleoside reverse transcriptase inhibitor (NNRTI): n = 30 (52.6%) Protease Inhibitor (PI): n = 39 (68.4%) Disease stage: Stage 1 = 59.6% [34/57] Stage 2 = 35.1% [20/57]) Stage 3 = 5.3% [3/57]
da Silva et al.,^5^ Santa Maria (RS), Brasil (2011)	Cross-sectional study	38 children and adolescents ♂;: 16 ♀;: 22	6 to 18 years	Viral charge: NR ART: Average duration 77 ± 41 months (range, 5.4-155.7 months; median, 76 months) Disease stage: NR

ART = antiretroviral therapy; SC = Santa Catarina; RS = Rio Grande do Sul; HIV = human immunodeficiency virus; CDC = Centers for Disease Control and Prevention;

♂ male sex;

♀ female sex;

NR = not reported;

* Information on authorship of this study.

Of the four included articles, three aimed to present the prevalence of body image dissatisfaction.^3–5^ In addition, the study by Alves Junior et al.^3^ also tested the association between body image and total fat mass, body composition (skinfolds), bone age, moderate to vigorous physical activity, viral load, and antiretroviral therapy.^3^ The study by Augustemak de Lima et al.^4^ tested the association between body image and body composition (body mass index [BMI], skinfolds, and circumferences), age, sex, and economic level.^4^ One study aimed at correlating body image (satisfied and dissatisfied) with self-esteem, physical activity, fat, and age^16^ (Table 2).

**Table 2:** Objectives, assessment instruments and forms of classification of body image, statistical analyzes, and results found in studies on body image in children and adolescents diagnosed with human immunodeficiency virus (HIV) infection

Author/Year	Study objectives	Body image assessment instrument/Body image classification form	Statistical analysis	Results of prevalence of body image dissatisfaction	Results found Correlations and/or factors associated with body image dissatisfaction
“AIDS among children--United States, 1996. ^16^ Division of HIV/AIDS Prevention, CDC”, New York, Florida, New Jersey, California, Puerto Rico and Texas - United States (1997)	Summarizing the Epidemiology of AIDS in Children in the United States reported from 1982 to 1996.	Dusek's short form of the Secord-Jourard Body Cathexis Scale (1983) Classification Body image: Answers one-two were classified as “I don't like my body,” three were classified as “neutral,” and four and five as “positive feelings”	Descriptive statistics Pearson correlation Multiple regression	NR	Dissatisfaction with body image was correlated with low self-esteem, low level of physical activity, and higher body fat when compared to the group satisfied with body image.
Alves Junior et al.,^3^ 2021	Check for differences in body fat values assessed by different methods according to the perception of body image of HIV-infected children and adolescents.	Silhouette Scale previously validated with adolescents from Florianópolis (Adami et al.,^11^ 2012). Body image classification: Satisfied (zero score); Want to reduce body weight (negative values); Want to increase body weight (positive values).	Covariance analysis	**Male** (P = 0.861 cohen-D = 0.579): Satisfied (n = 12/40.0%) Want to reduce body weight (n = 07/23.3%) Want to increase body weight (n = 11/36.7%) **Female** (P = 0.861 cohen-D = 0.579): Satisfied (n = 14/40.0%) Want to reduce body weight (n = 10/28.6%) Want to increase body weight (n = 11/31.4%)	**Male** There were no significant differences in body fat and body image indicators. **Female** Dissatisfaction with body image was associated with higher rates of trunk fat, total fat mass, and leg fat mass in relation to those satisfied with body image.
Augustemak de Lima et al.,^4^ 2018	Verify possible associations of anthropometric indicators, infection/treatment, sexual maturity, and sociodemographic characteristics with body image in adolescents living with HIV.	Silhouette Scale previously validated with adolescents from Florianópolis (Adami et al.,^11^ 2012) Body Image Classification: Satisfied; Want to reduce body weight; Want to increase body weight.	Chi-square test and Fisher's exact test Student's t test Mann-Whitney U Test Multiple linear regression	**Male (P = 0.009):** Satisfied (40.0%) Want to reduce body weight (8.0%) Want to increase body weight (52.0%) **Female (P = 0.285):** Satisfied (50.0%) Want to reduce body weight (21.9%) Want to increase body weight (28.1%)	Body image dissatisfaction was associated with female sex, older age, higher body weight, higher BMI, and greater arm muscle area in both sexes in relation to those satisfied with their body image.
da Silva et al.,^5^ 2011	Check the prevalence of image satisfaction body of children and adolescents with HIV/AIDS, using HAART.	Silhouette scale validated by Kakeshita et al.^15^ (2009) Body Image Classification: Satisfied with body image; Dissatisfied with thinness; Dissatisfied with being overweight.	Chi-square test Student's t test	**Total:** Satisfied with their body image (n = 6/15.8%) Dissatisfied with thinness (n = 16/42.1%) Dissatisfied with being overweight (n = 16/42.1%) **Male:** Satisfied with their body image (n = 03/18.8%) Dissatisfied with thinness (n = 08/50.0%) Dissatisfied with being overweight (n = 05/31.2%) **Female:** Satisfied with their body image (n = 03/13.6%) Dissatisfied with thinness (n = 08/36.4%) Dissatisfied with being overweight (n = 11/50.0%) **Children (age: NR):** Satisfied with their body image (n = 02/11.1%) Dissatisfied with thinness (n = 07/38.9%) Dissatisfied with overweight (n = 9/50.0%) **Adolescents (age: NR):** Satisfied with their body image (n = 04/20.0%) Dissatisfied with thinness (n = 09/45.0%) Dissatisfied with overweight (n = 07/35.0%)	Dissatisfaction with body image was associated with higher BMI and triceps skinfold in both sexes in relation to those satisfied with body image.

AIDS = acquired immunodeficiency syndrome; HAART = highly active antiretroviral therapy; BMI = body mass index; NR = not reported.

Regarding the instruments used to assess body image and the form of classification, two studies used the silhouette scale previously validated with adolescents from Florianópolis, Brazil,^17^ in which children and adolescents were classified as “satisfied with their body image”, “want to reduce body weight”, or “want to increase body weight”.^3,4^ One study used the Dusek's Secord-Jourard Body Cathexis Scale (1983), in which body image was classified using scores that resulted in three categories: “I do not like my body”, “neutral”, and “positive feelings”.^16,18^ One study used the silhouette scale validated by Kakeshita et al.,^19^ classifying children and adolescents as “satisfied with their body image”, “dissatisfied due to overweight”, or “dissatisfied due to thinness”^5^ (Table 2).

Regarding the results of articles found through this systematic review, it was found in three studies that children and adolescents of both sexes diagnosed with HIV infection were dissatisfied with their body image.^3–5^ The prevalence of body image dissatisfaction due to thinness ranged from 36.7%^3^ to 52.0%^4^ for males and from 28.1%^4^ to 36.4%^5^ for females. The prevalence of body image dissatisfaction due to overweight ranged from 8.0%^4^ to 31.2%^5^ for males and from 21.9%^4^ to 50.0%^5^ for females (Table 2).

The factors associated with body image dissatisfaction found in this review were: low levels of physical activity, higher body fat and low self-esteem,^16^ greater body weight, greater arm muscle area, greater triceps skinfold thickness ^5^, greater BMI,^4^ and being older and female,^4^ when compared to groups satisfied with their body image. Furthermore, body image dissatisfaction in females was associated with higher trunk fat, total fat mass, and leg fat mass in relation to those satisfied with their body image^3^ (Table 2).

We found that of the four cross-sectional studies included in this review, one did not present clear information about the individuals that composed the population and sample, participation rate of eligible individuals, inclusion/exclusion criteria, justification for the sample size, and sampling power description or estimates of variance and effect, as well as a lack of previous measurement of the exposure variable and no detailed description of them.^16^ Another study did not provide justification for the sample size, sample power description, or estimates of variance and effect, as well as a lack of previous measurement of the exposure variable.^5^ The other studies (n = 2) did not evaluate the exposure variable before measuring the result or provide enough time to verify the effect of associations^3,4^ (Table 3).

**Table 3:** Assessment of methodological quality in cross-sectional studies on body image in children and adolescents diagnosed with human immunodeficiency virus (HIV) infection

Author(s), year	Q1	Q2	Q3	Q4	Q5	Q6	Q7	Q8	Q9	Q10	Q11	Q12	Q13	Q14	Score Total
“AIDS among children-United States, 1996.^16^ Division of HIV/AIDS Prevention, CDC”, 1997	Y	N	NR	N	N	N	N	Y	Y	NA	Y	NA	NA	Y	08
Alves junior et al.,^3^ 2021	Y	Y	Y	Y	Y	N	N	Y	Y	NA	Y	NA	NA	Y	12
Augustemak de Lima et al.,^4^ 2018.	Y	Y	Y	Y	Y	N	N	Y	Y	NA	Y	NA	NA	Y	12
da Silva et al.,^5^ 2011	Y	Y	NR	Y	N	N	N	Y	N	NA	Y	NA	NA	NA	09

Y = yes; N = no; NA = not applicable; NR = not reported. 1- Was the research question or objective in this article clearly stated? 2- Was the study population clearly specified and defined? 3- Was the participation rate of eligible people at least 50%? 4- Were all subjects selected or recruited from the same or similar populations (including the same time period)? Were the inclusion and exclusion criteria for participating in the study pre-specified and applied uniformly to all participants? 5- Was a justification for the sample size, description of power, or estimates of variation and effect provided? 6- For the analyzes in this document, were the exposures of interest measured before the result(s) were measured? 7- Was the time frame long enough to reasonably expect to see an association between exposure and outcome, if any? 8- For exposures that may vary in amount or level, did the study examine different exposure levels in relation to outcome (eg. exposure categories or exposure measured as a continuous variable)? 9- Were exposure measures (independent variables) clearly defined, valid, reliable, and consistently implemented in all study participants? 10- Have the exposure(s) been evaluated more than once over time? 11- Were the outcome measures (dependent variables) clearly defined, valid, reliable, and consistently implemented in all study participants? 12- Were the outcome assessors blinded to the exposure status of the participants? 13- Was the loss to follow-up after the start of the study 20% or less? 14- The main potential confounding variables were measured and statistically adjusted for their impact on the relationship between exposure(s) and outcome(s).

## DISCUSSION

According to the full reading of the articles included in this systematic review,^3–5,16^ children and adolescents diagnosed with HIV infection reported body image dissatisfaction due to both excess weight and thinness. The highest prevalence identified in both sexes was in relation to dissatisfaction due to thinness. In addition, factors associated with body image dissatisfaction were low levels of physical activity, higher body fat, and low self-esteem,^16^ greater body weight, greater arm muscle area, greater triceps skinfold,^5^ greater BMI,^4,5^ in addition to being older and female.^4^ Furthermore, body image dissatisfaction in females was associated with higher trunk fat, total fat mass, and leg fat mass.^3^

The fact that children and adolescents diagnosed with HIV infection of both sexes were dissatisfied due to thinness (they would like to increase their body weight) can be explained, in part, by the weight loss found in children and adolescents diagnosed with the infection. HIV infection has a direct effect on the inhibition of human growth hormone (hGH) synthesis.^20^ Dissatisfaction due to thinness is recurrent mainly in male adolescents, due to the desire to have a stronger and more robust body.^4,21^ However, in a study of this review, this type of dissatisfaction was more frequently reported by females.^4^

In addition, children and adolescents in this systematic review also showed dissatisfaction due to excess weight (would like to reduce body weight), but these prevalences were not higher than those reported in relation to dissatisfaction due to thinness. This can be explained by the negative effects of the mass media on body image perception, as body image dissatisfaction is strongly related to standards imposed by society and culture.^22,23^ Thus, the increase in globalization and exposure to the ideal body (thin for females and muscular for males) through the media creates an even greater internal conflict in children and adolescentes.^23^ The articles included showed that body image dissatisfaction in children and adolescents diagnosed with HIV infection can be explained by the visible manifestations of HIV and the adverse effects of the treatment, such as weight loss and reduced muscle mass.^3–5,16^

Although studies included in the present review reported body image dissatisfaction in both sexes, this dissatisfaction differs from children and adolescents without an HIV infection diagnosis.^22,24–25^ While in the present systematic review, the highest prevalence of body image dissatisfaction reported was due to thinness, the literature shows that in female children and adolescents without HIV infection diagnosis, body image dissatisfaction is more recurrent due to overweight.^22,24–25^ Females in general want to reduce their body silhouette^22,24–25^ and not increase it, as verified in this systematic review for females with HIV infection diagnosis. This difference in relation to results can be explained by the stigma related to HIV infection and the continuous use of medication.^20^ These studies,^22,24–25^ used the silhouette scales validated by Adami et al.^17^ and the silhouette scale proposed by Kakeshita et al.^19^ to assess body image, as well as studies included in the present systematic review.

Regarding associated factors, disorders related to body image are associated with low self-esteem and changes in body composition, which can be explained because children and adolescents with high body mass have difficulties in relation to self-acceptance, consequently reporting low self-esteem.^3–5,16^ In addition, changes that occur during the transition period between childhood and adolescence, such as increase in body fat in the premenarche period, can generate body image dissatisfaction in females;^22,23^ anthropometric and body composition changes are observed by the marked development of lean body mass and muscle mass, which are factors that can generate body image dissatisfaction in males.^25,26^

In relation to associated factors, the loss of a child's role and identity in both sexes can generate body image dissatisfaction.^25,26^ In an analysis of body image dissatisfaction in several European countries with children and adolescents without a diagnosis of HIV infection, it was reported that high BMI values were associated with body image dissatisfaction.^26^ On the other hand, studies with children and adolescents without diagnosis of HIV infection and with adequate BMI reported body image dissatisfaction.^28–29^

Of the studies included in the present systematic review, only one verified the presence of lipodystrophy and its association with body image;^5^ however, it did not identify significant association for the sample. This can be explained by the assumption that infected individuals follow the same pattern of body image dissatisfaction as healthy individuals in the age group evaluated.^5^ In addition, although changes in body fat are noticeable to the evaluating physician, in children and adolescents, such changes are not as evident as in adults; this may not be perceived as a generator of body image dissatisfaction in children and adolescents with an HIV diagnosis.^5^ However, it is noteworthy that this study, considered a pioneer in Brazil in the assessment of body image in children and adolescents diagnosed with HIV infection using antiretroviral therapy, has limitations, such as lack of a control group, sample size, and time of data collection, which can impact the results.^5^ In the long term, adult individuals diagnosed with HIV infection have high levels of body image dissatisfaction, resulting in worse quality of affective and social relationships with friends and family.

The different protocols found in this review can be explained by the gradual increase in scientific production related to body image and the need for adequate instruments to assess certain age groups (children, adolescents, adults, and older adults).^30^ All studies included in this systematic review used assessment methods validated for use in children and adolescents in general. This systematic review showed that there are no specific validated instruments to assess body image of children and adolescents diagnosed with HIV infection, which opens an opportunity for further studies. Furthermore, the use of silhouette scales^17,19^ and others such as the Dusek's Secord-Jourard Body Cathexis Scale^18^ to measure body image is recurrent among researchers who investigate body image and body dissatisfaction in children and adolescentes.^17^ Assessments using full-body silhouette scales^17,19^ and body regions and functions scales^31^ are related to the attitudinal component, that is, they aim to measure the individuals' ability to perceive their own body dimension.^32^ The methods used to assess body image consider that the body image perception is not a mere challenge to see well, but to capture and interpret what is seen according to the body identity of each individual.^19^ However, the different instruments used to assess body image make further comparisons between studies difficult.^30^

It is important to mention the fact that of studies included in the present review, one carried out in the United States^7^ and the others in southern Brazil.^3–5^ The cultural differences of each country can influence the results obtained in each study.^33^ It is inevitable that each individual internalizes a set of beliefs, attitudes, values, and behaviors, which are transmitted from generation to generation and common to all individuals in a given culture.^34^

Regarding the results of the assessment of the risk of bias/methodological quality of studies, it was possible to identify that two studies reached a score of 12,^3,4^ one study 9^5^ and one 8.^16^ All had scores considered to be of moderate risk of bias/reasonable methodological quality.^12^ This means that studies are susceptible to some bias errors, but such errors are considered insufficient to invalidate the results.^15^ As a characteristic of studies with reasonable methodological quality, variation was identified in relation to strengths and limitations.^15^ The assessment of the risk of bias/methodological quality of studies is a tool that helps reviewers to focus on concepts that are fundamental to the internal validity of each study.^15^

Among the limitations of this review, the cross-sectional design of all included studies should be highlighted, which does not allow temporal or causal relationships. Furthermore, due to the small number of studies, it was not possible to carry out more in-depth analyses regarding the differences between sexes and age groups, and the different instruments used to assess body image make the comparison of results difficult. Another limitation identified by the review was the fact that there is a nearly four decades of difference between the United States study, which reported cumulative data from 1982 to 1996,^16^ and the other three studies included in the present review.^3–5^ This time difference between studies makes it difficult to generalize the findings because, over four decades, cultural characteristics and the body perception and relationship may change as a result of the dynamics of society. Furthermore, HIV treatment has made significant advances that have improved patients' quality of life over these four decades.

As positive aspects of this study, the pioneering of conducting a systematic review of body image in children and adolescents diagnosed with HIV infection stands out, adopting a search strategy in eight different databases. Lastly, it is suggested for future studies that the presence of children and adolescents with acquired immunodeficiency syndrome (AIDS) and not only those diagnosed with HIV infection be identified in samples to better understand the influence of AIDS on body image and associated factors.

## CONCLUSIONS

In conclusion, the findings of this systematic review show that children and adolescents of both sexes diagnosed with HIV infection are dissatisfied with their body image. Regarding factors associated with body image, low levels of physical activity, greater body fat, low self-esteem, greater body weight, greater arm muscle area, greater triceps skinfold, and greater BMI were identified in both sexes and are associated with body image dissatisfaction. There is no consensus on how body image is assessed, given the variety of instruments identified in this review, which demonstrates the need for monitoring and developing interventions aimed at reducing body image dissatisfaction.

## Appendix 1 Keywords

**Table:**

1 - PubMed via National Library of Medicine (MEDLINE) search performed on October 6, 2021.		
Bloc	Descriptors	Articles
1	*“body image” OR “self perception” OR “self image” OR “body satisfaction” OR “body,* *dissatisfaction” OR “self esteem” OR “body perception” OR “weight perception”*	***All fields:*** 52,279 ***Title/abstract:*** 42,425
1 + 2	*child* OR adolec* OR student OR youth OR adolescent* OR adolescence OR teen OR teenage OR teenager OR scholar OR “young people” OR “school children” OR “school teenager” OR young OR childhood OR “children with HIV” OR “adolescents with HIV” OR “children infected with HIV” OR “adolescents infected with HIV” OR “HIV children” OR “HIV adolescents” OR “children living with HIV” OR “adolescents living with HIV”*	***All fields:*** 29,294 ***Title/abstract:*** 15,491
1 + 2 + 3	*HIV OR AIDS OR “Human Immunodeficiency Virus” OR “Acquired Immunodeficiency Syndrome”*	***All fields:*** 671 ***Title/abstract:*** 380
((“*body image*” OR “*self perception*” OR “*self image*” OR “*body satisfaction*” OR “*body dissatisfaction*” OR “*self esteem*” OR “*body perception*” OR “*weight perception*”) AND (*child** OR *adolec** OR *student* OR *youth* OR *adolescent** OR *adolescence* OR *teen* OR *teenage* OR *teenager* OR *scholar* OR “*young people*” OR “*school children*” OR “*school teenager*” OR *young* OR *childhood* OR “*children with HIV*” OR “*adolescents with HIV*” OR “*children infected with HIV*” OR “*adolescents infected with HIV*” OR “*HIV children*” OR “*HIV adolescents*” OR “*children living with HIV*” OR “*adolescents living with HIV*”)) AND (HIV OR AIDS OR “*Human Immunodeficiency Virus*” OR “*Acquired Immunodeficiency Syndrome*”) *Filters applied: Female, Male, Child: birth-18 years, Preschool Child: 2-5 years, Child: 6-12 years, Adolescent: 13-18 years*		405

2 - Web of Science search performed on: October 6, 2021.		
Bloc	Descriptors	Articles
1	*“body image” OR “self perception” OR “self image” OR “body satisfaction” OR “body dissatisfaction” OR “self esteem” OR “body perception” OR “weight perception”*	***All fields:*** 76,697 ***Title:*** 20,625
1 + 2	*child* OR adolec* OR student OR youth OR adolescent* OR adolescence OR teen OR teenage OR teenager OR scholar OR “young people” OR “school children” OR “school teenager” OR young OR childhood OR “children with HIV” OR “adolescents with HIV” OR “children infected with HIV” OR “adolescents infected with HIV” OR “HIV children” OR “HIV adolescents” OR “children living with HIV” OR “adolescents living with HIV”*	***All fields:*** 38,708 ***Title:*** 6,041
1 + 2 + 3	*HIV OR AIDS OR “Human Immunodeficiency Virus” OR “Acquired Immunodeficiency Syndrome”*	***All fields:*** 907 ***Title:*** 24
*You searched for: ALL FIELDS: (“body image*” OR “*self perception*” OR “*self image*” OR “*body satisfaction*” OR “*body dissatisfaction*” OR “*self esteem*” OR “*body perception*” OR “*weight perception*”) *AND ALL FIELDS*: (*child** OR *adolec** OR *student* OR *youth* OR *adolescent** OR *adolescence* OR *teen* OR *teenage* OR *teenager* OR *scholar* OR “*young people*” OR “*school children*” OR “*school teenager*” OR *young* OR *childhood* OR “*children with HIV*” OR “*adolescents with HIV*” OR “*children infected with HIV*” OR “*adolescents infected with HIV*” OR “*HIV children*”		465

3 - Scopus search performed on October 6, 2021.		
Bloc	Descriptors	Articles
1	*“body image” OR “self perception” OR “self image” OR “body satisfaction” OR “body dissatisfaction” OR “self esteem” OR “body perception” OR “weight perception”*	***All fields:*** 183,568 ***Title/abstract/keywords:*** 11,456
1 + 2	*child* OR adolec* OR student OR youth OR adolescent* OR adolescence OR teen OR teenage OR teenager OR scholar OR “young people” OR “school children” OR “school teenager” OR young OR childhood OR “children with HIV” OR “adolescents with HIV” OR “children infected with HIV” OR “adolescents infected with HIV” OR “HIV children” OR “HIV adolescents”* *OR “children living with HIV” OR “adolescents living with HIV”*	***All fields:*** 831 ***Title/abstract/keywords:*** 1
1 + 2 + 3	*HIV OR AIDS OR “Human Immunodeficiency Virus” OR “Acquired Immunodeficiency Syndrome”*	***All fields:*** 419 ***Title/abstract/keywords:*** 0
(ALL (“*body* AND *image*” OR “*self* AND *perception*” OR “*self* AND *image*” OR “*body* AND *satisfaction*” OR “*body* AND *dissatisfaction*” OR “*self* AND *esteem*” OR “*body* AND *perception*” OR “*weight* AND *perception*”) AND ALL (*child** O R *adolec** OR *student* OR *youth* OR *adolescent** OR *adolescence* OR *teen* OR *t eenage* OR *teenager* OR *scholar* OR “*young* AND *people*” OR “*school* AND *child ren*” OR “*school* AND *teenager*” OR *young* OR *childhood* OR “*children* AND *wit h* AND *hiv*” OR “*adolescents* AND *with* AND *hiv*” OR “*children* AND *infected* A ND *with* AND *hiv*” OR “*adolescents* AND *infected* AND *with* AND *hiv*” OR “*hiv* AND *children*” OR “*hiv* AND *adolescents*” OR “*children* AND *living* AND *with* A ND *hiv*” OR “*adolescents* AND *living* AND *with* AND *hiv*”) AND ALL (*hiv* OR *aids* OR “*human* AND *immunodeficiency* AND *virus*” OR “*acquired* AND *immuno deficiency* AND *syndrome*”)) AND (EXCLUDE (SUBJAREA, “BIOC”) OR EX CLUDE (SUBJAREA, “IMMU”) OR EXCLUDE (SUBJAREA, “ARTS”) OR E XCLUDE (SUBJAREA, “AGRI”) OR EXCLUDE (SUBJAREA, “DENT”) OR EXCLUDE (SUBJAREA, “PHAR”) OR EXCLUDE (SUBJAREA, “ENGI”) OR EXCLUDE (SUBJAREA, “ENVI”) OR EXCLUDE (SUBJAREA, “CENG”) OR EXCLUDE (SUBJAREA, “BUSI”) OR EXCLUDE (SUBJAREA, “EART”) OR EXCLUDE (SUBJAREA, “ECON”) OR EXCLUDE (SUBJAREA, “VETE”) OR EXCLUDE (SUBJAREA, “CHEM”) OR EXCLUDE (SUBJAREA, “COMP”) O R EXCLUDE (SUBJAREA, “MATH”))		286

4 - SPORTDiscus via EBSCOhost search performed on October 6, 2021.		
Bloc	Descriptors	Articles
1	*“body image” OR “self perception” OR “self image” OR “body satisfaction” OR “body dissatisfaction” OR “self esteem” OR “body perception” OR “weight perception”*	***Full text:*** 38,567 ***Title:*** 2,905 ***Abstract:*** 6,665
1 + 2	*child* OR adolec* OR student OR youth OR adolescent* OR adolescence OR teen OR teenage OR teenager OR scholar OR “young people” OR “school children” OR “school teenager” OR young OR childhood OR “children with HIV” OR “adolescents with HIV” OR “children infected with HIV” OR “adolescents infected with HIV” OR “HIV children” OR “HIV adolescents” OR “children living with HIV” OR “adolescents living with HIV”*	***Full text:*** 30,515 ***Title:*** 886 ***Abstract:*** 2,998
1 + 2 + 3	*HIV OR AIDS OR “Human Immunodeficiency Virus” OR “Acquired Immunodeficiency Syndrome”*	***Full text:*** 3.075 ***Title:*** 02 ***Abstract:*** 18
TX (“*body image*” OR “*self perception*” OR “*self image*” OR “*body satisfaction*” OR “*body dissatisfaction*” OR “*self esteem*” OR “*body perception*” OR “*weight perception*”) AND TX (*child** OR *adolec** OR *student* OR *youth* OR *adolescent** OR *adolescence* OR *teen* OR *teenage* OR *teenager* OR *scholar* OR “*young people*” OR “*school children*” OR “*school teenager*” OR *young* OR *childhood* OR “*children with HIV*” OR “*adolescents with HIV*” OR “*children infected with HIV*” OR “*adolescents infected with HIV*” OR “*HIV children*” OR “*HIV adolescents*” OR “*children living with HIV*” OR “*adolescents living with HIV*”) AND TX (HIV OR AIDS OR “*Human Immunodeficiency Virus*” OR “*Acquired Immunodeficiency Syndrome*”) Restringir por *SubjectThesaurus*: *psychology* , *psychological stress* , *health* , *health behavior* , *physical education* , *quality of life* , *attitude (psychology)* , *public health* , *self-perception* , *mental depression* , *physical activity* , *mental health* , *college students* , *physical fitness* , *medical care* , *health promotion* , *risk-taking behavior* , *therapeutics* , *hiv infections* , *motivation (psychology)* , *exercise* , *self-evaluation* , *self-efficacy* , *teenagers* , *psychological tests* , *body image* , *hiv-positive persons* , *sports* , *well-being* , *emotions* , *sports psychology* , *anxiety* , *athletes* , *health education* , *hiv* , *self-esteem* , *patients* , *eating disorders* , *education* , *personality* , *aids* , *nutrition* , *diseases* , *distress (psychology)* , *universities & colleges* , *health status indicators* , *decision making* , *adolescent psychology* , *body mass index* , *perception* , *health attitudes* , *cognition* , *leisure* , *sexually transmitted dise…* , *women* , *mental illness* , *social psychology* , *teenagers’ health* , *women's health* , *disease risk factors* , *obesity* , *youth* , *motor ability* , *psychology of college stu…* , *treatment effectiveness* , *children* , *college athletes* , *diet* , *physiology* , *resilience (personality t…* , *child development* , *chronic diseases* , *dietary supplements* , *gender identity* , *human sexuality* , *prevention* , *medical personnel* , *body weight* , *pathological psychology* , *lifestyles* , *undergraduates* , *aids patients* , *intention* , *physical education teache…* , *physical training & condi…* , *sports participation* , *educational attainment* , *evidence-based medicine* , *life skills* , *school children* , *weight loss* , *young adults* , *body composition* , *children's health* , *diagnosis* , *ethics* , *high school students* , *medical screening* , *recreation* , *food habits* , *psychology of women* , *sports sciences* , *stress management* , *student attitudes* , *exercise therapy* , *recreational therapy* , *sex education* , *sports personnel* , *students* , *symptoms* , *child psychology* , *childhood obesity* , *cognitive therapy* , *college student attitudes* , *goal (psychology)* , *health self-care* , *regulation of body weight* , *teachers* *sports psychology* , *anxiety* , *athletes* , *health education* , *hiv* , *self-esteem* , *patients* , *eating disorders* , *education* , *personality* , *aids* , *nutrition* , *diseases* , *distress (psychology)* , *universities & colleges* , *health status indicators* , *decision making* , *adolescent psychology* , *body mass index* , *perception* , *health attitudes* , *cognition* , *leisure* , *sexually transmitted dise…* , *women* , *mental illness* , *social psychology* , *teenagers’ health* , *women's health* , *disease risk factors* , *obesity* , *youth* , *motor ability* , *psychology of college stu…* , *treatment effectiveness* , *children* , *college athletes* , *diet* , *physiology* , *resilience (personality t…* , *child development* , *chronic diseases* , *dietary supplements* , *gender identity* , *human sexuality* , *prevention* , *medical personnel* , *body weight* , *pathological psychology* , *lifestyles* , *undergraduates* , *aids patients* , *intention* , *physical education teache…* , *physical training & condi…* , *sports participation* , *educational attainment* , *evidence-based medicine* , *life skills* , *school children* , *weight loss* , *young adults* , *body composition* , *children's health* , *diagnosis* , *ethics* , *high school students* , *medical screening* , *recreation* , *food habits* , *psychology of women* , *sports sciences* , *stress management* , *student attitudes* , *exercise therapy* , *recreational therapy* , *sex education* , *sports personnel* , *students* , *symptoms* , *child psychology* , *childhood obesity* , *cognitive therapy* , *college student attitudes* , *goal (psychology)* , *health self-care* , *regulation of body weight* , *teachers*		507

5 - LILACS via Biblioteca Virtual em Saúde search performed on October 6, 2021.		
Bloc	Descriptors	Articles
1	*body image* OR *self perception* OR *self image* OR *body satisfaction* OR *body dissatisfaction* OR *self esteem* OR *body perception* OR *weight perception*	***Words:*** 2,567 ***Title:*** 00 ***Abstract:*** 00
1 + 2	*child$* OR *adolec$* OR *student* OR *youth* OR *adolescent$* OR *adolescence* OR *teen* OR *teenage* OR *teenager* OR *scholar* OR *young people* OR *school children* OR *school teenager* OR *young* OR *childhood* OR *children with HIV* OR *adolescents with HIV* OR *children infected with HIV* OR *adolescents infected with HIV* OR *HIV children* OR *HIV adolescents* OR *children living with HIV* OR *adolescents living with HIV*	***Words:*** 00 ***Title:*** 00 ***Abstract:*** 00
1 + 2 + 3	HIV OR AIDS OR *Human Immunodeficiency Virus* OR *Acquired Immunodeficiency Syndrome*	***Words:*** 21 ***Title:*** 00 ***Abstract:*** 0
*body image* OR *self perception* OR *self image* OR *body satisfaction* OR *body dissatisfaction* OR *self esteem* OR *body perception* OR *weight perception* [*words*] and *child*$ OR *adolec*$ OR *student* OR *youth* OR *adolescent$* OR *adolescence* OR *teen* OR *teenage* OR *teenager* OR *scholar* OR *young people* OR *school children* OR *school teenager* OR *young* OR *childhood* OR *children with HIV* OR *adolescents with HIV* OR *children infected with HIV* OR *adolescents infected with HIV* OR *HIV children* OR *HIV adolescents* OR *children living with HIV* OR *adolescents living with HIV* [*words*] and HIV OR AIDS OR *Human Immunodeficiency Virus* OR *Acquired Immunodeficiency Syndrome* [*words*]		21
1	*Imagen corporal OR autopercepción OR autoimagen OR satisfacción corporal OR insatisfacción corporal OR autoestima OR percepción corporal OR percepción del peso*	***Words:*** 4,306 ***Title:*** 462 ***Abstract:*** 2,134
1 + 2	*niño$ OR adolescente$ OR estudiante OR joven OR adolescencia OR escolar OR jóvenes OR niños en edad escolar OR adolescente en edad escolar OR joven OR niñez OR niños con VIH OR adolescentes con VIH OR niños infectados con el VIH OR adolescentes infectados con el VIH OR niños con VIH OR adolescentes con VIH OR niños que viven con el VIH OR adolescentes que viven con el VIH*	***Words:*** 1,823 ***Title:*** 104 ***Abstract:*** 621
1 + 2 + 3	*VIH OR SIDA OR Virus de inmunodeficiencia humana OR Síndrome de inmunodeficiencia adquirida*	***Words:*** 35 ***Title:*** 00 ***Abstract*** *:* 11
*Imagen corporal* OR *autopercepción* OR *autoimagen* OR *satisfacción corporal* OR *insatisfacción corporal* OR *autoestima* OR *percepción corporal* OR *percepción del peso* [*Words*] AND *niño$* OR *adolescente$* OR *estudiante* OR *joven* OR *adolescencia* OR *escolar* OR *jóvenes* OR *niños en edad escolar* OR *adolescente en edad escolar* OR *joven* OR *niñez* OR *niños con VIH* OR *adolescentes con VIH* OR *niños infectados con el VIH* OR *adolescentes infectados con el VIH* OR *niños con VIH* OR *adolescentes con VIH* OR *niños que viven con el VIH* OR *adolescentes que viven con el VIH* [*Words*] AND VIH OR SIDA OR *Virus de inmunodeficiencia humana* OR *Síndrome de inmunodeficiencia adquirida* [*Words*]		35

6 - Scientific Eletronic Library Online (SciELO) search performed on October 6, 2021.		
Bloc	Descriptors	Articles
1	“*body image*” OR “*self perception*” OR “*self image*” OR “*body satisfaction*” OR “*body dissatisfaction*” OR “*self esteem*” OR “*body perception*” OR “*weight perception*”	***All fields:*** 961 ***Abstract:*** 821 ***Title:*** 131
1 + 2	*child* OR adolec* OR student OR youth OR adolescent* OR adolescence OR teen OR teenage OR teenager OR scholar OR “young people” OR “school children” OR “school teenager” OR young OR childhood OR “children with HIV” OR “adolescents with HIV” OR “children infected with HIV” OR “adolescents infected with HIV” OR “HIV children” OR “HIV adolescents” OR “children living with HIV” OR “adolescents living with HIV”*	***All fields:*** 00 ***Title:*** 00 ***Abstract:*** 00
1 + 2 + 3	HIV OR AIDS OR “*Human Immunodeficiency Virus*” OR “*Acquired Immunodeficiency Syndrome*”	***All fields:*** 00 ***Title:*** 00 ***Abstract:*** 00
(“*body image*” OR “*self perception*” OR “*self image*” OR “*body satisfaction*” OR “*body dissatisfaction*” OR “*self esteem*” OR “*body perception*” OR “*weight perception*”) AND (*child* OR adolec* OR student OR youth OR adolescent* OR adolescence OR teen OR teenage OR teenager OR scholar OR “young people” OR “school children” OR “school teenager” OR young OR childhood OR “children with HIV” OR “adolescents with HIV” OR “children infected with HIV” OR “adolescents infected with HIV” OR “HIV children” OR “HIV adolescents” OR “children living with HIV” OR “adolescents living with HIV”) AND (*HIV OR AIDS OR “Human Immunodeficiency Virus” OR “Acquired Immunodeficiency Syndrome”)		00
1	*“Imagen corporal” OR “autopercepción” OR “autoimagen” OR “satisfacción corporal” OR “insatisfacción corporal” OR “autoestima” OR “percepción corporal” OR “percepción del peso”*	***Todos los índices:*** 2,228 ***Resumen:*** 2,011 ***Título:*** 585
1 + 2	*niño* OR adolescente* OR estudiante OR joven OR adolescencia OR escolar OR jóvenes OR “niños en edad escolar” OR “adolescente en edad escolar” OR joven OR niñez OR “niños con VIH” OR “adolescentes con VIH” OR “niños infectados con el VIH” OR “adolescentes infectados con el VIH” OR “niños con VIH” OR “adolescentes con VIH” OR “niños que viven con el VIH” OR “adolescentes que viven con el VIH”*	***Todos los índices:*** 00 ***Resumen:*** 00 ***Título:*** 00
1 + 2 + 3	VIH OR SIDA OR “*Virus de inmunodeficiencia humana*” OR “*Síndrome de inmunodeficiencia adquirida*”	***Todos los índices:*** 00 ***Resumen:*** 00 ***Título:*** 00
(“*Imagen corporal*” OR “*autopercepción*” OR “*autoimagen*” OR “*satisfacción corporal*” OR “*insatisfacción corporal*” OR “*autoestima*” OR “*percepción corporal*” OR “*percepción del peso*”) AND (*niño** OR *adolescente** OR *estudiante* OR *joven* OR *adolescencia* OR *escolar* OR *jóvenes* OR “*niños en edad escolar*” OR “*adolescente en edad escolar*” OR *joven* OR *niñez* OR “*niños con VIH*” OR “*adolescentes con VIH*” OR “*niños infectados con el VIH*” OR “*adolescentes infectados con el VIH*” OR “*niños con VIH*” OR “*adolescentes con VIH*” OR “*niños que viven con el VIH*” OR “*adolescentes que viven con el VIH*”) AND (VIH OR SIDA OR “*Virus de inmunodeficiencia humana*” OR “*Síndrome de inmunodeficiencia adquirida*”)		00

7 - PsycINFO via American Psychological Association (APA) search performed on October 6, 2021.		
Bloc	Descriptors	Articles
1	*“body image” OR “self perception” OR “self image” OR “body satisfaction” OR “body dissatisfaction” OR “self esteem” OR “body perception” OR “weight perception”*	***All fields:*** 103,817 ***Title:*** 21,034 ***Abstract:*** 63,778
1 + 2	*child* OR adolec* OR student OR youth OR adolescent* OR adolescence OR teen OR teenage OR teenager OR scholar OR “young people” OR “school children” OR “school teenager” OR young OR childhood OR “children with HIV” OR “adolescents with HIV” OR “children infected with HIV” OR “adolescents infected with HIV” OR “HIV children” OR “HIV adolescents”* *OR “children living with HIV” OR “adolescents living with HIV”*	***All fields:*** 58,888 ***Title:*** 5,062 ***Abstract:*** 24,447
1 + 2 + 3	*HIV OR AIDS OR “Human Immunodeficiency Virus” OR “Acquired Immunodeficiency Syndrome”*	***All fields:*** 986 ***Title:*** 20 ***Abstract*** *:* 342
“*body image*” OR “*self perception*” OR “*self image*” OR “*body satisfaction*” OR “*body dissatisfaction*” OR “*self esteem*” OR “*body perception*” OR “*weight perception*” *AND **Any Field:** child** OR *adolec** OR *student* OR *youth* OR *adolescent** OR *adolescence* OR *teen* OR *teenage* OR *teenager* OR *scholar* OR “*young people*” OR “*school children*” OR “*school teenager*” OR *young* OR *childhood* OR “*children with HIV*” OR “*adolescents with HIV*” OR “*children infected with HIV*” OR “*adolescents infected with HIV*” OR “*HIV children*” OR “*HIV adolescents*” OR “*children living with HIV*” OR “*adolescents living with HIV*” *AND **Any Field:*** HIV OR AIDS OR “*Human Immunodeficiency Virus*” OR “*Acquired Immunodeficiency Syndrome*” *AND **Publication Type:** Peer Reviewed Journal AND **Age Group:** Adolescence (13-17 yrs) AND **Age Group:** Childhood (birth-12 yrs) AND **Age Group:** School Age (6-12 yrs) AND **Age Group:** Preschool Age (2-5 yrs)*		08

8 - Cumulative Index to Nursing and Allied Health Literature (CINAHL) via EBSCOhost search performed on October 6, 2021.		
Bloc	Descriptors	Articles
1	*“body image” OR “self perception” OR “self image” OR “body satisfaction” OR “body dissatisfaction” OR “self esteem” OR “body perception” OR “weight perception”*	***Full texts:*** 66,194 ***Title:*** 7,224 ***Abstract:*** 21,517
1 + 2	*child* OR adolec* OR student OR youth OR adolescent* OR adolescence OR teen OR teenage OR teenager OR scholar OR “young people” OR “school children” OR “school teenager” OR young OR childhood OR “children with HIV” OR “adolescents with HIV” OR “children infected with HIV” OR “adolescents* *infected with HIV” OR “HIV children” OR “HIV adolescents” OR “children living with HIV” OR “adolescents living with HIV”*	***Full texts:*** 47,812 ***Title:*** 2,285 ***Abstract:*** 9,056
1 + 2 + 3	*HIV OR AIDS OR “Human Immunodeficiency Virus” OR “Acquired Immunodeficiency Syndrome”* TX (“*body image*” OR “*self perception*” OR “*self image*” OR “*body satisfaction*” OR “*body dissatisfaction*” OR “*self esteem*” OR “*body perception*” OR “*weight perception*”) AND TX (*child** OR *adolec** OR *student* OR *youth* OR *adolescent** OR *adolescence* OR *teen* OR *teenage* OR *teenager* OR *scholar* OR “*young people*” OR “*school children*” OR “*school teenager*” OR *young* OR *childhood* OR “*children with HIV*” OR “*adolescents with HIV*” OR “*children infected with HIV*” OR “*adolescents infected with HIV*” OR “*HIV children*” OR “*HIV adolescents*” OR “*children living with HIV*” OR “*adolescents living with HIV*”) AND TX (HIV OR AIDS OR “*Human Immunodeficiency Virus*” OR “*Acquired Immunodeficiency Syndrome*”) ***Restringir por SubjectAge:*** - *all infant, child: 6-12 years, adolescent: 13-18 years, all child, child, preschool: 2-5 years*. ***Restringir por SubjectMajor:*** ***Restringir por SubjectMajor:*** *hiv infections* , *sexuality* , *quality of life* , *hiv- positive persons* , *risk taking behavior* , *stigma* , *support* , *psychosocial* , *black persons* , *depression* , *health behavior* , *self concept* , *women* , *adaptation* , *psychological* , *stress* , *psychological* , *women's health* , *health promotion* , *mental health* , *chronic disease* , *students* , *college* , *mental disorders* , *interpersonal relations* , *psychological well-being* , *life experiences* , *patient attitudes* , *sex education* , *acquired immunodeficiency…* , *gay men* , *adolescent behavior* , *health services accessibi…* , *health status* , *communication* , *attitude to sexuality* , *attitude to health* , *self care* , *hispanic americans* , *body image* , *sexually transmitted dise…* , *palliative care* , *sexual health* , *parent-child relations* , *coping* , *hardiness* , *adolescent health* , *caregivers* , *health knowledge* , *decision making* , *homosexuality* , *truth disclosure* , *attitude to aids* , *lgbtq± persons* , *perception* , *health education* , *self-efficacy* , *students* , *high school* , *adolescent psychology* , *student attitudes* , *students* , *attitude* , *family relations* , *anxiety* , *hiv seropositivity* , *motivation* , *pediatric obesity* , *counseling* , *disease transmission*	***Full texts:*** 7,016 ***Title:*** 19 ***Abstract:*** 209
